# Can Aspirin Use Be Associated With the Risk or Prognosis of Bladder Cancer? A Case-Control Study and Meta-analytic Assessment

**DOI:** 10.3389/fonc.2021.633462

**Published:** 2021-07-19

**Authors:** Bo Fan, Alradhi Mohammed, Yuanbin Huang, Hong Luo, Hongxian Zhang, Shenghua Tao, Weijiao Xu, Qian Liu, Tao He, Huidan Jin, Mengfan Sun, Man Sun, Zhifei Yun, Rui Zhao, Guoyu Wu, Xiancheng Li

**Affiliations:** ^1^ Department of Urology, Second Affiliated Hospital of Dalian Medical University, Dalian, China; ^2^ Clinical Medicine, Dalian Medical University, Dalian, China; ^3^ Medical Imaging, Dalian Medical University, Dalian, China; ^4^ Department of Anaesthesiology, Fifth Affiliated Hospital of Sun Yat-Sen University, Zhuhai, China; ^5^ Department of Pharmacy, Zhongshan College of Dalian Medical University, Dalian, China; ^6^ Department of Pharmacy, First Affiliated Hospital of Dalian Medical University, Dalian, China

**Keywords:** urinary bladder neoplasms, aspirin, risk, prognosis, case-control, meta-analysis

## Abstract

Aspirin, widely used to prevent cardiovascular disease, had been linked to the incidence of bladder cancer (BCa). Existing studies focusing on Chinese populations are relatively rare, especially for Northeast China. Meanwhile, relevant studies on the effects of aspirin on the occurrence or prognosis of BCa are inconsistent or even controversial. First, in the case control study, logistic regression analysis was used to investigate the association between aspirin intake and risk of BCa including 1121 patients with BCa and the 2242 controls. Subsequently, Kaplan-Meier curve and Cox regression analyses were applied to explore the association between aspirin intake and clinicopathological factors which may predict overall survival (OS) and recurrence-free survival (RFS) of BCa patients. Finally, we quantificationally combined the results with those from the published literature evaluating aspirin intake and its effects on the occurrence, outcome of surgery and prognosis of BCa by meta-analysis up to May 1, 2021.Our case-control study demonstrated that the regular use of aspirin was not associated with a reduced incidence of BCa (*P*=0.175). Stratified analyses of sex showed that aspirin intake did not lead to a lower risk of BCa in female patients (*P*=0.063). However, the male population who regularly took aspirin had a lower incidence of BCa (OR=0.748, 95% CI= 0.584-0.958, *P*=0.021). Subgroup analyses stratified by smoking found a significant reduction in the risk of BCa in current smokers with aspirin intake (OR=0.522, 95% CI=0.342-0.797, *P*=0.002). In terms of prognosis of BCa, patients with a history of aspirin intake did not had a markedly longer OS or RFS than those with no history of aspirin intake by Kaplan-Meier curves. Stratified analysis by sex showed no correlation between aspirin intake and the recurrence or survival of BCa for either male or female patients. However, in people younger than 68, aspirin intake seemed to have prolonged effects for overall survival (HR=3.876; 95% CI=1.326-11.325, *P*=0.019). Then, we performed a meta-analysis and the combined results from 19 articles and our study involving more than 39524 BCa cases indicated that aspirin intake was not associated with the occurrence of BCa (*P*=0.671). Subgroup analysis by whether regular use of aspirin, by the mean duration of use of aspirin, by sex, by smoking exposure, by research region and by study type also supported the above results. In terms of the impact of aspirin intake on the prognosis of patients with BCa, 11 articles and our study involving 8825 BCa cases were eligible. The combined results showed that patients with aspirin intake did not have significantly influence on survival, recurrence, progression and metastasis than those without aspirin intake. On the whole, both our retrospective study and literature meta-analysis suggested a lack of a strong relevant association between the use of aspirin and the incidence or prognosis of BCa. Thus, additional long-term follow-up prospective research is warranted to clarify the association of aspirin with BCa incidence and prognosis.

## Background

Bladder cancer (BCa) is the 11th most common cancer in the world. The global age-standardized incidence rate per 100,000 persons/year is 2.2 for women and 9.0 for men (1, 2). According to the National Cancer Institute, the estimated numbers of new BCa cases and deaths in the USA alone (2014) will be 74,690 and 15,580, respectively (3, 4). In China, the incidence and mortality rates have increased gradually in the past few years. According to the National Central Cancer Registry (NCCR) of China 2015 annual report, the overall incidence of BCa was 7.68/10^5^ (5, 6). Among patients with superficial or non-muscle invasive tumors after transurethral resection or perfusion therapy, 70% would experience recurrence, and 10% to 20% would show progression to muscle-invasive tumors (7, 8). Due to the unfavorable prognosis of muscle-invasive cancer, the treatment involves multiple modalities, including radical surgery, radiotherapy, and chemotherapy. However, nearly half of these patients develop metastases and die within 3 years (7, 9). The occurrence or recurrence of BCa is a molecular biological change or process that is effected by occupational factors, non-occupational factors, and genomics and proteomics factors (10) Other non-occupational factors, including cigarette smoking (11), drinking water used for washing or cleaning drinking water used for washing or cleaning (12) the consumption of substances with nitrate and nitrite content (13), alcohol consumption (14), and special drug intake (15, 16), have also been associated with BCa but are less well established. Therefore, early detection strategies and prognosis monitoring are essential for reducing mortality from BCa.

Aspirin, a typical non-steroidal anti-inflammatory drug, has been widely used for pain, fever and cardiovascular disease (17, 18) In recent years, a large accounts of studies have suggested that aspirin has a potential preventive effect in several types of cancers (18–20). The antitumor activity of aspirin is thought to be based mainly on two different mechanisms. First, aspirin may interfere with carcinogenesis by inhibiting the target of cyclooxygenase (COX), which is produced in response to inflammation and leads to angiogenesis and reduced apoptosis. As one of isoforms of cyclooxygenase, the level of COX-2 may not only increase the malignant properties of BCa cells but also be related to high-grade and advanced-stage BCa patients (21, 22). Second, aspirin plays roles in promoting apoptosis or inhibiting the proliferation of tumor cells by interfering with the pathway independent of COX-2 as an anticancer agent. Aspirin inhibits the growth of PI3K mutant breast cancer by activating AMP-activated protein kinase (AMPK) and inhibiting mechanistic target of rapamycin complex 1 (mTORC1) signaling independent of COX-2 and IKK-β/NF-κB (23). Furthermore, aspirin inhibited the proliferation of neuroblastoma cells, upregulated p21Waf1 and regulated Rb1 to promote differentiation through a Cox-independent mechanism (24).

Since aspirin has apparent preventive effects on other tumors, its role in BCa has also received attention. The findings of the impact of aspirin intake and the incidence and mortality of BCa have been inconclusive. Moreover, few studies on the relationship between aspirin intake and BCa in mainland China, especially in Northeast China. To explore whether the use of aspirin is associated with altered risk of BCa, we conducted a case-control study by enrolling 1121 patients with BCa. To avoid bias from single-center study results, we further completed a meta-analysis of eligible literature by searching electronic journals or databases for documents before March 1, 2021. The connection between aspirin intake and the incidence and prognosis of BCa was investigated, which helps to further determine whether the use of aspirin has a preventive effect on BCa. Our analysis provides a basis for further exploration of the role of aspirin in the future.

## Materials and Methods

### Retrospective Study

#### Study Population for Exploring the Association of Aspirin Intake and Risk of Bladder Cancer

This study was approved by the institutional review committee of the Second Affiliated Hospital of Dalian Medical University. The study population consisted of 1121 bladder cancer cases with average age 68 years old, ranging from 49 to 84 years. The control group comprised of 2242 individuals without a bladder cancer diagnosis during the study period, which were hospitalized in the department of respiratory, gastroenterology, orthopedic surgery, dermatology, and cardiology, etc.

Patients in both groups underwent 1:2 matching based on sex, age, and follow-up period at the index date of each case. The possible risk factors for the occurrence of BCa, including age, marital status, smoking status, alcohol use, aspirin use, metformin use, history of cardiovascular disease, history of cerebrovascular disease and history of diabetes were extracted from clinical history information.

#### Study Population for Exploring Association of Aspirin Intake and Prognosis of Bladder Cancer

1121 patients in the case group who were pathologically diagnosed with bladder cancer by pathological biopsy or surgical biopsy at the Second Affiliated Hospital of Dalian Medical University were included in the study. The clinical and pathological baseline data, including age, gender, histological grading, T stage, lymph node metastasis, distant metastasis, type of surgery were recorded. TMN staging and histological grading were determined according to the 2004 WHO/ISUP classification. Recurrence of bladder cancer is defined as visual and/or biopsy evidence of a tumor confirmed by cystoscopy or urine cytology.

#### Assessment of Aspirin Use

Aspirin users were defined as those who used at least 81mg aspirin daily at least twice a week for 1 month or more. Nonusers are subjects who had never used the drug or had used it for <1 month. The average daily dose of aspirin for each aspirin user was also obtained by dividing the cumulative dose of aspirin by the cumulative number of days of aspirin use. The indication for the use of aspirin (i.e., analgesic or cardiovascular disease, cerebrovascular disease prevention) was also recorded (25–27). Patients were stratified according to aspirin intake for most (50% or more) of the interval between diagnosis and the date of the first tumor recurrence or last follow-up. The exclusion criteria were as follows: (1). Unable to obtain accurate medication records, demographic data, or patient characteristics. (2). Identification of a history of exposure to carcinogens. (3). Patients on clopidogrel, warfarin, or statins alone or in combination with other fibrin clotting inhibitors. (4). Aspirin use was contraindicated.

#### Statistical Analysis

SPSS version 13.0 (SPSS Inc., Chicago, USA) was used for analysis. The associations between aspirin intake and clinicopathological parameters were evaluated by the chi-square test. Continuous data and frequency data were analyzed by t test and Fisher’s exact test. When the independent variables were screened in order to investigate the risk factors for the occurrence of BCa, adjusted odds ratios (ORs) or relative risks (RRs) and the corresponding 95% confidence intervals (CIs) were calculated by univariate and multivariate logistic regression analysis. By univariate analysis, those with a significant *P* value less than 0.05 in this test were included in the multivariate regression analysis. To evaluate the overall survival (OS) which was the interval from surgery to death and recurrence-free survival (RFS) which was the interval from surgery to intravesical recurrence in patients with BCa, the Kaplan-Meier curve and log-rank test were used. After assessing clinicopathologic factors by univariate Cox regression model, statistically significant variables were put into multivariate Cox regression analysis to find independent prognostic factors for OS and RFS. After SPSS analysis, the *P* value of the above parameters was obtained. A *P*-value less than 0.05 was considered statistically significant.

### Meta-Analysis

#### Search Strategy

The implementation of this meta-analysis was conducted based on the “Preferred Reporting Items for Systematic Reviews and Meta-Analyses” (PRISMA) guidelines. The PRISMA checklist was shown in **Table 1**. Our meta-analysis has been already registered of the review protocol on PROSPERO (CRD42021245411). The study retrieved articles from the PubMed, Embase, Ovid Medicine, Cochrane Library and Scopus databases published before May 1, 2021, to identify relevant studies evaluating aspirin intake and its effects on the occurrence and prognosis of BCa using the following medical subject headings that include all spelling variations: “bladder cancer” and ”aspirin”, “occurrence”, “risk” and “prognosis”, “survival” and “recurrence”. Without national and linguistic restrictions, after reviewing the duplicate data, the two reviewers independently screened the titles and abstracts, excluding articles that were not associated with our research, reviews, and related animal experiments and maximizing data quality.

**Table 1:** PRISMA checklist.

Section/topic	#	Checklist item	Reported on page #
**TITLE**			
Title	1	Identify the report as a systematic review, meta-analysis, or both.	1
**ABSTRACT**			
Structured summary	2	Provide a structured summary including, as applicable: background; objectives; data sources; study eligibility criteria, participants, and interventions; study appraisal and synthesis methods; results; limitations; conclusions and implications of key findings; systematic review registration number.	1-2
**INTRODUCTION**			
Rationale	3	Describe the rationale for the review in the context of what is already known.	2
Objectives	4	Provide an explicit statement of questions being addressed with reference to participants, interventions, comparisons, outcomes, and study design (PICOS).	2
**METHODS**			
Protocol and registration	5	Indicate if a review protocol exists, if and where it can be accessed (e.g., Web address), and, if available, provide registration information including registration number.	3
Eligibility criteria	6	Specify study characteristics (e.g., PICOS, length of follow-up) and report characteristics (e.g., years considered, language, publication status) used as criteria for eligibility, giving rationale.	3
Information sources	7	Describe all information sources (e.g., databases with dates of coverage, contact with study authors to identify additional studies) in the search and date last searched.	3
Search	8	Present full electronic search strategy for at least one database, including any limits used, such that it could be repeated.	3
Study selection	9	State the process for selecting studies (i.e., screening, eligibility, included in systematic review, and, if applicable, included in the meta-analysis).	3
Data collection process	10	Describe method of data extraction from reports (e.g., piloted forms, independently, in duplicate) and any processes for obtaining and confirming data from investigators.	3
Data items	11	List and define all variables for which data were sought (e.g., PICOS, funding sources) and any assumptions and simplifications made.	3
Risk of bias in individual studies	12	Describe methods used for assessing risk of bias of individual studies (including specification of whether this was done at the study or outcome level), and how this information is to be used in any data synthesis.	5; **Figure 1**
Summary measures	13	State the principal summary measures (e.g., risk ratio, difference in means).	5
Synthesis of results	14	Describe the methods of handling data and combining results of studies, if done, including measures of consistency (e.g., I^2^) for each meta-analysis.	5
Risk of bias across studies	15	Specify any assessment of risk of bias that may affect the cumulative evidence (e.g., publication bias, selective reporting within studies).	5
Additional analyses	16	Describe methods of additional analyses (e.g., sensitivity or subgroup analyses, meta-regression), if done, indicating which were pre-specified.	5
**RESULTS**			
Study selection	17	Give numbers of studies screened, assessed for eligibility, and included in the review, with reasons for exclusions at each stage, ideally with a flow diagram.	8; **Figure 2**
Study characteristics	18	For each study, present characteristics for which data were extracted (e.g., study size, PICOS, follow-up period) and provide the citations.	8, 9; **Tables 7**–**9**
Risk of bias within studies	19	Present data on risk of bias of each study and, if available, any outcome level assessment (see item 12).	5; **Figure 1**
Results of individual studies	20	For all outcomes considered (benefits or harms), present, for each study: (a) simple summary data for each intervention group (b) effect estimates and confidence intervals, ideally with a forest plot.	9, 10, 12-17; **Figures 3**, **5**–**13**
Synthesis of results	21	Present results of each meta-analysis done, including confidence intervals and measures of consistency.	9, 10, 12-17; **Figures 3**, **5**–**13**
Risk of bias across studies	22	Present results of any assessment of risk of bias across studies (see Item 15).	9, 10, 12-17; **Figures 3**, **5**–**13**
Additional analysis	23	Give results of additional analyses, if done (e.g., sensitivity or subgroup analyses, meta-regression [see item 16]).	9, 10, 12-17; **Figure 4**
**DISCUSSION**			
Summary of evidence	24	Summarize the main findings including the strength of evidence for each main outcome; consider their relevance to key groups (e.g., healthcare providers, users, and policy makers).	18, 19
Limitations	25	Discuss limitations at study and outcome level (e.g., risk of bias), and at review-level (e.g., incomplete retrieval of identified research, reporting bias).	19
Conclusions	26	Provide a general interpretation of the results in the context of other evidence, and implications for future research.	19
**FUNDING**			
Funding	27	Describe sources of funding for the systematic review and other support (e.g., supply of data); role of funders for the systematic review.	20

#### Selection Criteria

Studies satisfying the following criteria were included in our analysis: (1) the histologic type of the tumors was urothelial carcinoma of the bladder by histologic or pathologic examination; (2) the association between aspirin intake and the risk of BCa or prognosis of patients with BCa was investigated; and (3) sufficiency of data for the calculations of OR/RR/hazard ratio (HR), 95% CI and *P*-value. Accordingly, the following exclusion criteria were applied: (1) studies in the form of reviews, letters to the editor, commentaries, or case reports that lacked original data; (2) molecular biology research that explored the impact of aspirin on cancer cell lines and animal models; and (3) studies in which the HR/OR/RR and its standard error could not be collected based on the given information.

#### Data Extraction

After the full-text evaluation, the two authors extracted the data separately for further qualitative and quantitative analysis to increase the authenticity of the data. For the selected articles, we extracted data from each study, including the first author, publication time, region, type of study, study period the number of participants and BCa cases, age, the usage of aspirin, HR/OR/RR, and adjustment factors.

#### Quality Assessment

**Figure 1:**
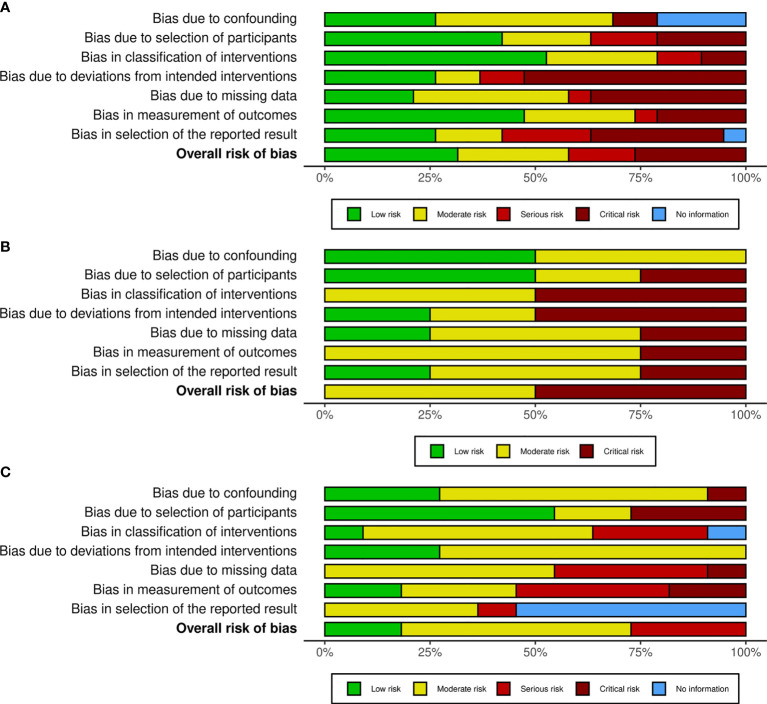
Methodological quality graph for meta-analysis of the incidence **(A)**, outcomes of surgery **(B)** and prognosis **(C)** of bladder cancer. The review authors’ judgements about each methodological quality item of ROBINS-I presented as different colors across all included studies. The red, yellow and green colors represent critical, moderate and low bias, respectively.

The quality assessment used the most recent version of the risk of bias tool recommended in the ROBINS-I checklist (28) for systematic reviews of interventions for the included studies. Our selected studies were assessed for bias due to confounding, selection bias, intervention measurement, missing data bias, outcome measurement, reporting bias, and other types of bias. As shown in **Figure 1**, the risk of bias of our meta-analyses measured by ROBINS-I framework for individual studies was generally low to moderate.

#### Sensitivity Analysis

Whether influential studies needed to be secondary for exclusion from our meta-analysis was determined by sensitivity analysis. The robustness of the pooled estimates with respect to the employed individual investigations was evaluated using the leave-one-out method based on the Q-test. Lower heterogeneity was determined after eliminating the indicated studies if the *P*_Q_ value > 0.05. Conversely, low heterogeneity was calculated by removing a single study, indicating that the selected studies did not contribute to the heterogeneity of the overall meta-analysis.

#### Statistical Analysis

The Stata 12.0 statistical software package (Stata Corp, College Station, TX, USA) was used for all data analyses. We calculated the OR/RR/HR with a 95% CI. The Q-test was used to assess the statistical heterogeneity and judge the p-value qualitatively. The I^2^ value in the I^2^ test describes the proportion of the total variation due to heterogeneity rather than sampling errors, and I^2^> 50% or *P*<0.05 indicates a high degree of heterogeneity. The random-effects model was used; otherwise, there was no heterogeneity, and the fixed-effects model was used. The potential bias was assessed by a funnel plot and Egger’s test, and when the funnel plot was symmetrically distributed, there was no significant bias. Conversely, if the funnel plot exhibits skewness and asymmetry, this indicated bias.

## Results

### Retrospective Study to Explore the Association of Aspirin Intake and Risk of Bladder Cancer

#### Baseline Characteristics

The clinical characteristics of the 1121 cases and 2242 controls are shown in **Table 2**. The following variable information was selected as the prognostic factors in our study: age at diagnosis <68 or >68, marital status, smoking status (nonsmoker, current smoker, former smoker), alcohol use (nondrinker, current drinker, former drinker), history of aspirin intake (yes, no), history of metformin use (yes, no), history of cardiovascular disease (yes, no), history of cerebrovascular disease (yes, no), and history of diabetes (yes, no). In the multivariate logistic regression analysis, we found that gender (OR=1.263; 95%CI=1.026-1.556; *P*=0.028); marital status(OR=0.165; 95%CI=0.940-0.289; *P*<0.001); current smoking status (OR=2.360; 95%CI=1.936-2.875; *P*<0.001); current alcohol use (OR=0.615; 95%CI=0.496-0.762; *P*<0.001); former alcohol use(OR=0.314; 95%CI=0.188-0.525; *P*<0.001) and history of cardiovascular disease (OR=0.212; 95%CI=0.162-0.276; *P*<0.001) were associated with a strong risk of BCa.

**Table 2:** Demographic characteristics of bladder cancer cases and controls.

	Case group (n = 1121)	Control group (n = 2242)	Total	*P* value
Gender				**0.002**
Female	193	487	680	
Male	928	1755	2683	
Asprin use				0.181
No	1008	1981	2989	
Yes	113	261	374	
Age, years				0.661
<68	562	1142	1704	
≥ 68	559	1100	1659	
Marriage				**<0.001**
No	55	19	74	
Yes	1066	2223	3289	
Smoking				**<0.001**
Non-smoker	609	1390	1999	
Current smoker	449	531	980	
Former smoker	63	321	384	
Alcohol use				**<0.001**
Non-drinker	861	1631	2492	
Current drinker	239	478	717	
Former drinker	21	133	154	
History of cardiovascular disease				**0.002**
No	1025	1970	2995	
Yes	96	272	368	
History of diabetes				**<0.001**
No	928	1736	2664	
Yes	193	506	699	
History of cerebrovascular disease				**<0.001**
No	1049	1661	2710	
Yes	72	581	653	
Metformin use				0.177
No	1067	2108	3175	
Yes	54	134	188	

The bold values were applied to highlight P-values which had statistically significance (i.e. P<0.05).

#### The Association of Aspirin Intake and Risk of Bladder Cancer

Among the controls and the cases identified as BCa, the patients who used aspirin accounted for 10.1% of the total number of patients with BCa and 11.6% of the control group. As shown in **Table 3**, The analysis showed that the regular use of aspirin was not associated with a reduced incidence of BCa (OR=0.851, 95%CI= 0.674-1.075, *P*=0.175). Subgroup analyses stratified by sex showed no correlation between aspirin intake and the risk of BCa in female patients (OR=2.000, 95% CI=0.950-4.190, *P*=0.063). However, the male population who regularly took aspirin had a lower incidence of BCa (OR=0.748, 95% CI= 0.584-0.958, *P*=0.021). Moreover, subgroup analyses stratified by smoking status showed no reduction in the risk of BCa for nonsmokers (OR=1.200, 95% CI=0.879-1.630, *P*=0.252) or former smokers (OR=0.913, 95% CI=0.437-1.900, *P*=0.807) who used aspirin daily. In contrast, our results found a significant reduction in the risk of BCa in current smokers with aspirin intake (OR=0.522, 95% CI=0.342-0.797, *P*=0.002). In addition, subgroup analyses stratified by drinking status showed no change in the risk of BCa for nondrinkers (OR=0.970, 95% CI=0.744-1.260, *P*=0.822), current drinkers (OR=0.651, 95% CI=0.377-1.120, *P*=0.121), or former drinkers (OR=0.227, 95% CI= 0.029-1.780, *P*=0.126) who used aspirin daily.

**Table 3:** Univariate and multivariate logistic regression analysis of risk factors for bladder cancer.

	Univariate analysis		Multivariate analysis	
	HR (95%CI)	p value	HR (95%CI)	*P* value
Sex Female vs. Male	1.334 (1.109-1.605)	**0.002**	1.263 (1.026-1.556)	**0.028**
Aspirin use Yes vs. no	0. 851 (0.674-1.075)	0.175		
Age <68 vs. >68	1.033 (0.895-1.192)	0.661		
Marital Status Yes vs. Others	0.166 (0.098-0.280)	**<0.001**	0.165 (0.94-0.289)	**<0.001**
Smoking				
Non-smoker				
Current smoker	1.930 (1.648-2.260)	**<0.001**	2.360 (1.936-2.875)	**<0.001**
Former smoker	0.448 (0.336-0.596)	**<0.001**	0.744 (0.537-1.031)	0.076
Alcohol use				
Non-drinker				
Current drinker	0.947 (0.794-1.129)	0.545	0.615 (0.496-0.762)	**<0.001**
Former drinker	0.299 (0.187-0.477)	**<0.001**	0.314 (0.188-0.525)	**<0.001**
History of cerebrovascular disease yes vs. others	0.678 (0.531-0.866)	**0.002**	0.863 (0.662-1.126)	0.279
diabetes yes vs. others	0.714 (0.594-0.858)	**<0.001**	0.845 (0.694-1.028)	0.093
History of cardiovascular disease yes vs. others	0.196 (0.152-0.254)	**<0.001**	0.212 (0.162-0.276)	**<0.001**
Metformin use yes vs. others	0.796 (0.576-1.101)	0.168		

The bold values were applied to highlight P-values which had statistically significance (i.e. P<0.05).

### Retrospective Study to Explore the Association of Aspirin Intake and Prognosis of Bladder Cancer

#### Aspirin Intake and Clinicopathological Characteristics of Bladder Cancer

A total of 1121 patients with newly developed BCa from Northeast China, mainland China, were identified from 2002 to January 2019 and divided into aspirin and non-aspirin groups. Of these patients in **Table 4**, the male to female ratio was 928:193, and there were 559 patients aged 68 or greater. A total of 344 patients (30.7%) had pathologic stage T_2_-T_4_ disease, and 687 patients (61.3%) had high-grade disease (G2 to G3). Positive lymph nodes were present in 62 patients (5.3%), and distant metastasis was present in 33 patients (2.9%). All patients had a definite type of surgery; 854 underwent TURBT (76.2%), 58 underwent partial resection (5.2%), and 209 underwent radical prostatectomy (18.6%). The characteristics of bladder cancer patients, such as sex (*P*=0.090) and type of surgery (*P*=0.172), were not correlated with a history of aspirin intake. Similarly, tumor characteristics such as pT stage (*P*=0.475), pathologic grade (*P*=0.852), lymph node status (*P*=0.866), and distant metastasis (*P*=0.693) were also not correlated with a history of aspirin intake. However, age (*P*<0.001) was associated with a history of aspirin intake.

**Table 4:** Association between aspirin intake and clinico-pathological characteristics of 1121 BCa patients.

	Regular Aspirin use		Total	*P* value
	No	Yes		
Gender				0.090
Male	828	100	928	
Female	180	13	193	
Age, years				**<0.001**
Less than 68	529	33	562	
68 or Greater	479	80	559	
pT stage				0.475
T_is_-T_1_	702	75	777	
T_2_-T_4_	306	38	344	
Pathologic grade				0.852
G_1_	393	41	434	
G_2_	165	19	184	
G_3_	450	53	503	
Lymph node status				0.866
No	951	108	1059	
N_1_	37	4	41	
N_2_	18	1	19	
N_3_	2	0	2	
Distant metastasis				0.693
M_0_	979	109	1088	
M_1_	29	4	33	
Type of surgery				0.172
TURBT	773	81	854	
PC	54	4	58	
RC	181	28	209	

TURBT, Transurethral resection of bladder tumor; PC, Partial cystectomy; RC, Radical cystectomy. The bold values were applied to highlight P-values which had statistically significance (i.e. P<0.05).

#### The Association of Aspirin Intake and the Prognosis in Patients With BCa

Of the 1121 patients, the median follow-up among survivors was 50.1 months. Seventy-nine patients (7.0%) died, and 235 patients (21.0%) experienced relapse. By KM plotter drawing, we did not find that patients with a history of aspirin intake had a markedly longer OS or RFS than those with no history of aspirin intake. To explore prognostic factors for bladder cancer, patient-specific and tumor-specific features, together with a history of aspirin intake, were incorporated into the Cox regression model. Notably, distant metastasis (HR=2.611; 95% CI=1.170-5.826, *P*=0.019) and type of surgery (HR=1.398; 95% CI=1.066-1.833, *P*=0.015) were significantly associated with OS (**Table 5**). Moreover, pT stage (HR=1.728; 95% CI=1.324-2.254, *P*<0.001) was an independent predictor for RFS after multivariate analysis. However, a history of aspirin intake was not an independent factor for OS or RFS (**Table 6**). Stratified analysis by sex showed no correlation between aspirin intake and the recurrence of BCa for either male patients (*P*=0.325) or female patients (*P*=0.617). Furthermore, aspirin intake was not significantly associated with overall survival in male (*P*=0.071) or female patients (*P*=0.646) with BCa. Stratified analysis by sex showed no correlation between aspirin intake and the recurrence of BCa for either male patients (*P*=0.325) or female patients (*P*=0.617). Stratified analysis by age showed no change between aspirin intake and the recurrence of BCa for both patients younger than 68 years old (*P*=0.489) and patients older than 68 years (*P*=0.916). Furthermore, aspirin intake was not significantly associated with overall survival in patients older than 68 years (*P*=0.318). Interestingly, aspirin intake (HR=3.876; 95% CI=1.326-11.325, *P*=0.019) and type of surgery (HR=1.685; 95% CI=1.131-1.131, *P*=0.010) were significantly associated with OS in BCa patients younger than 68 years old after Cox multivariate regression analysis.

**Table 5:** Univariate and multivariate Cox regression model for overall survival (OS) including known parameters in 1121 BCa patients treated with surgery.

Overall Survival	Univariate analysis		Multivariate analysis	
	HR (95%CI)	*P* value	HR (95%CI)	*P* value
Gender	2.344 (1.018–5.397)	**0.045**	2.137 (0.924-4.944)	0.076
Age	1.697 (1.077–2.675)	**0.023**	1.392 (0.869-2.229)	0.169
pT stage	1.810 (1.157–2.832)	**0.009**	1.218 (0.728-2.037)	0.453
Pathologic grade	1.201 (0.939–1.535)	0.145		
Lymph node status	0.662(0.273–1.606)	0.362		
Distant metastasis	3.552 (1.626-7.760)	**0.001**	2.611 (1.170-5.826)	**0.019**
Type of surgery	1.598 (1.263-2.023)	**<0.001**	1.398 (1.066-1.833)	**0.015**
Aspirin use	2.045 (1.100–3.802)	**0.024**	1.653 (0.871-3.137)	0.124

The bold values were applied to highlight P-values which had statistically significance (i.e. P<0.05).

**Table 6:** Univariate and multivariate Cox regression model for recurrence-free survival (RFS) including known parameters in 1121 BCa patients treated with surgery.

Overall Survival	Univariate analysis		Multivariate analysis	
	HR (95%CI)	*P* value	HR (95%CI)	*P* value
Gender	1.070 (0.753–1.521)	0.706		
Age	1.284 (0.992–1.663)	0.058		
pT stage	1.812 (1.396–2.351)	**<0.001**	1.728 (1.324-2.254)	**<0.001**
Pathologic grade	1.173 (1.016–1.353)	**0.029**	1.103 (0.954-1.227)	0.186
Lymph node status	1.321(0.960–1.819)	0.087		
Distant metastasis	1.936 (1.055-3.551)	**0.033**	1.702 (0.925-3.132)	0.087
Type of surgery	1.133 (0.971-1.321)	0.112		
Aspirin use	1.170 (0.772–1.774)	0.459		

The bold values were applied to highlight P-values which had statistically significance (i.e. P<0.05).

### Meta-Analysis

#### Study Identification and Selection

**Figure 2:**
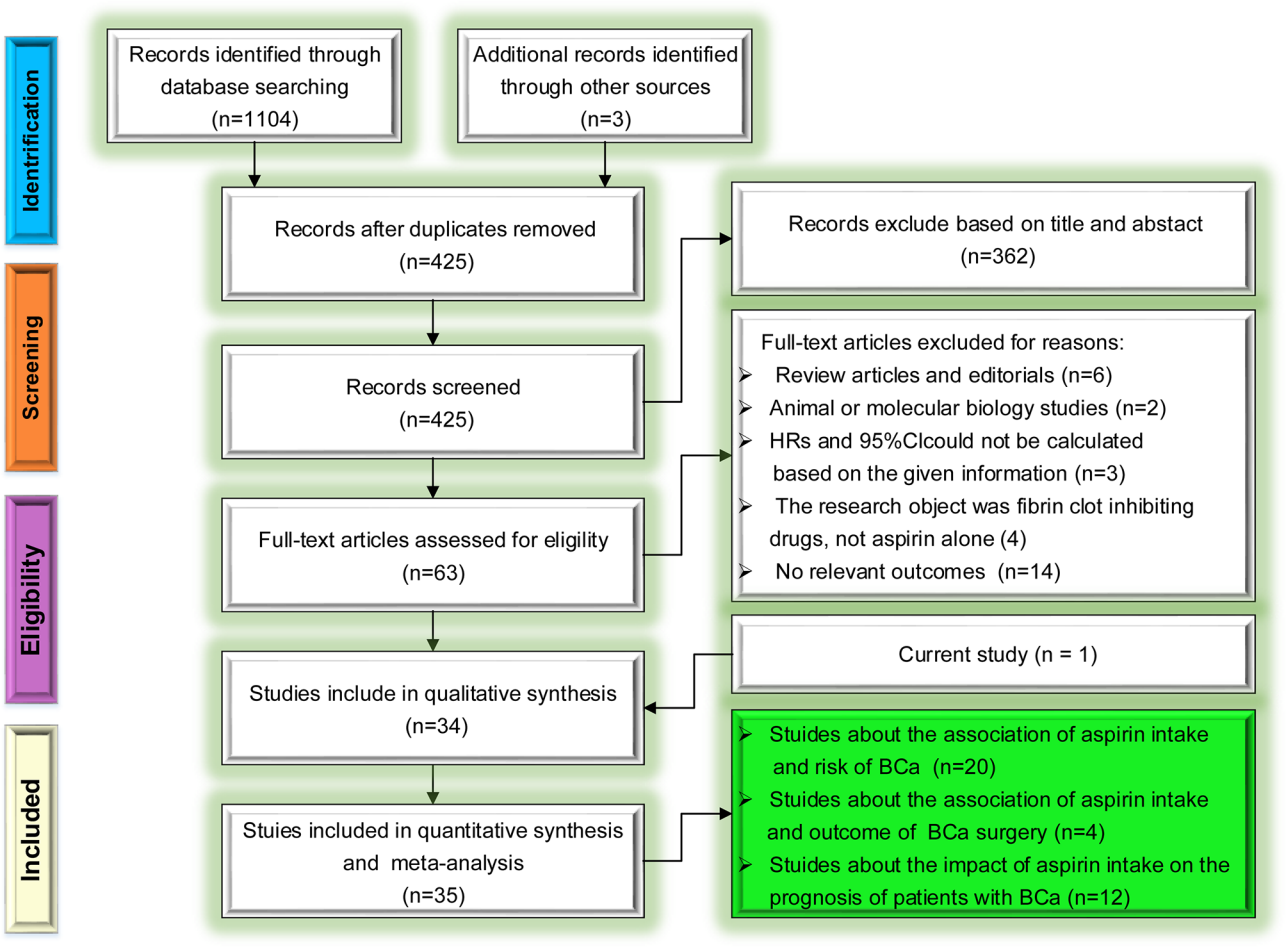
Preferred Reporting Items for Systematic Review and Meta-Analysis (PRISMA) flow diagram for the selection of articles.

The selection process following the Preferred Reporting Items for Systematic Review and Meta-Analysis (PRISMA) reporting guideline for the association of aspirin intake with the risk and prognosis of BCa is presented in **Figure 2**. Initially, the database search retrieved 1107 relevant publications. After screening the titles, abstracts and full texts of these articles, we excluded duplicate studies and other studies for various reasons (reviews/editorials, animal/molecular biology studies, or not relevant to our analysis etc.). Thus, in the final analysis, we included 19 studies about the relation of aspirin intake risk of BCa, 4 studies about outcome of BCa surgery and 11 studies about the relation of aspirin intake and prognosis of patients with BCa based on the inclusion criteria.

#### Characteristics of the Studies

The characteristics of the 19 included trials (29–47) about the relation of aspirin intake and risk of BCa were provided in **Table 7**. These trials were published between 1989 and 2021. Of the 20 included articles which include our studies, finally contains 31 studies. In these studies, nine were conducted in the North America (29–31, 33–36, 44, 45), four in Asia (38, 41, 42), four in Europe (32, 37, 46, 47), and three in multi-regions (39, 40, 43). Among all studies enrolled, eleven were cohort studies (29, 30, 34, 38–42, 44–46), and eight were case-control studies (31–33, 35–37, 47) containing this study. The given sample size of the trials ranged from 839 to 612509, with a total of more than 39524 patients with BCa. The characteristics of the four included trials (48–51) involving the relationship of aspirin intake and clinical course of bladder tumor surgery were provided in **Table 8**. These researches were published from 2013 to 2019 and from the USA (50), Italy (51), France (49) and Germany (48), respectively. The characteristics of the eleven included trials regarding the impact of aspirin intake on the prognosis of patients with BCa were presented in **Table 9**. The selected studies were published between 2004 and 2021. Combined with our study, eight studies originated from the USA (52, 53, 55–60), one was from Italy (61), one was from India (54) and one was from multi-regions (39). The sample size of the trials ranged from 43 to 139896. Among the twelve studies enrolled, eleven were cohort studies (39, 52–61), and only one was case-control study.

**Table 7:** Main characteristics of individual studies included in the meta-analysis on impact of aspirin intake on risk of BCa.

First author (year)	Region	Study type	Study period	Age	Total number	BCa Cases	Dose of use	Frequency/duration of use	Adjusted HR/RR/OR (95% CI)
Loomans-Kropp HA (39)	Multi-regions	Cohort study;Prospective	1993-2001	55-74	139896	1751	NA	≥3 times/week	0.99 (0.90-1.10)
								<3 times/week	0.96 (0.87-1.06)
Orchard SG (40)	Multi-regions	Cohort study; Prospective	2010-2014	≥ 65	19030	142	100mg/day	NA	1.02 (0.59-1.77)
Sung JJ (41)	China	Cohort study; Retrospective	2000-2013	>18	138966	2962	≥80mg/day	≥6 months	0.93 (0.83-1.05)
Tsoi K (38)	China	Cohort study; Retrospective	2000-2004	67.5	612509	5291	80mg/day	≥6 months	1.06 (0.98-1.14)
Guercio V (37)	Italy	Case control; Retrospective	2003-2014	67	1355	690	NA	≥1 times/week for ≥6 months	1.21 (0.87-1.68)
Kang M (42)	Korea	Cohort Study; Prospective	2002-2013	>40	320613	2121	NA	NA	1.05 (0.87-1.27)
Stegeman I (43)	Multi-regions	Others	2006-2015	40-85	NA	NA	NA	NA	0.94 (0.64-1.38)
Brasky TM (44)	USA	Cohort study; Prospective	1993-2010	50-79	126689	154	≤100mg/day	≥2 times/week for ≥2 weeks	1.12 (0.63-1.98)
Shih C (36)	USA	Case control; Retrospective	2000-2010	50-76	77048	385	Low use 81mg	1–3 days/week for <4 years	0.87 (0.64-1.18)
								4 days/week for ≥4 years	1.00 (0.73-1.38)
							Regular Aspirin NA	1–3 days/week for <4 years	1.00 (0.73-1.38)
								4 days/week for ≥4 years	1.03 (0.77-1.38)
Daugherty SE (35)	USA	Case control; Retrospective	1993-2001	63.5	508842	2489	NA	<2 times/week	1.03 (0.92-1.15)
								>2 times/week	1.04 (0.94-1.15)
Genkinger JM (34)	USA	Cohort study; Retrospective	1986-2002	40-75	49448	607	NA	≥2 times/week	0.99 (0.83–1.18)
Fortuny J (33)	USA	Case control; Retrospective	1997-2000	25-74	839	376	NA	≥ 4 times/week for ≥1 month	0.60 (0.40–0.90)
Jacobs EJ (45)	USA	Cohort Study; Prospective	1992-2003	≥50	146113	18127	≥325mg/day	Nonregular use	1.03 (0.86 - 1.24)
								<5 years:	0.97 (0.76 - 1.22)
								>5 years:	0.83 (0.58 -1.19)
Fortuny J (32)	Spain	Case control; Retrospective	1997-2000	20-80	1987	958	NA	Nonregular use	1.00 (0.80-1.20)
								≥ 2 times/week for ≥1 month	1.00 (0.70-1.50)
Friis S (46)	Denmark	Cohort study; Prospective	1989-1997	70	29470	134	75-150 mg/day	1 time/day for 3 months	1.20 (1.00-1.40)
Castelao JE (31)	USA	Case control; Retrospective	1987-1996	58	3028	1514	NA	≥ 2 times/week for ≥ 1 month	0.85 (0.66-1.09)
Pommor W (47)	Germany	Case control; Retrospective	1990-1995	NA	2180	571	NA	NA	1.09 (0.73-1.64)
Schreinemachers DM (30)	USA	Cohort study; Prospective	1982-1987	25-74	12668	35	NA	≥ 1 time/month	1.06 (0.54-2.09)
Paganini-Hill A (29)	USA	Cohort study; Prospective	1981-1988	73	13987	96	NA	< 1 time/day	Male: 0.37 (0.13-1.02) Female: 1.52 (0.55-4.23)
								≥ 1 time/day	Male: 1.12 (0.63-1.99) Female: 0.89 (0.26-3.10)
This study	China	Case control; Retrospective	2002-2019	68	3363	1121	≥80mg/day	≥1 months	0.85(0.67-1.08)

**Table 8:** Main characteristics of eligible studies collected in the meta-analysis on effect of aspirin intake on outcome of surgery for BCa.

First author (year)	Region	Study type	Study period	Age	BCa Cases	Surgical approach	Dose of use	Frequency/Duration of use	Adjusted HR/RR/OR (95% CI)
Wessels F (48)	Germany	Retrospective cohort study	2011-2017	65	461	RC	NA	NA	BTR:1.12(0.80-1.57) IACR:1.48 (0.77-2.86)
Prader R (49)	France	Retrospective case-control study	2013-2015	53-83	234	TURBT	≥75mg/day	NA	BTR:1.38 (0.09-21.70)
									IACR:1.15 (0.36-3.64)
									IHO:4.12 (0.17-99.83)
Ghali F (50)	USA	Retrospective case-control study	2011-2014	70	708	TURBT	NA	NA	IACR:0.38 (0.15-0.92)
Picozzi S (51)	Italy	Cohort study	2007-2012	74-68	158	TURBT	100mg/day	NA	BTR:1.94 (0.36-10.39)
									IHO:1.46 (0.25-8.55)

IACR, Incidence of any-cause rehospitalization; BTR, Blood transfusion rate; IHO, Incidence of hemostatic operation; TURBT, Transurethral resection of bladder tumor; RC, Radical cystectomy.

**Table 9:** Main characteristics of eligible studies collected in the meta-analysis on effect of aspirin intake on prognosis of BCa.

First author (year)	Region	Study type	Study period	Age	Total number	BCa cases	Dose of use	Frequency/ Duration of use	Reported endpoints
Loomans-Kropp HA (39)	Multi-regions	Cohort study; Prospective	1993-2001	65	139896	1751	NA	≥ 3 times/week	CSS: 0.67 (0.51-0.88)
								< 3 times/week	CSS:0.75 (0.58-0.98)
Li P (52)	USA	Cohort study; Retrospective	2016	65	63308	2600	NA	NA	OS: 1.01 (1.00-1.03)
Lyon TD (53)	USA	Cohort study; Retrospective	2007-2016	NA	1061	1061	25mg/day 81mg/day 162mg/day 325mg/day 650mg/day	3 months	CSS: 0.64 (0.45-0.89)
									OS: 0.70 (0.53-0.93)
									MFS: 0.96 (0.68-1.36)
Gupta R (54)	India	Cohort study; Prospective	2015-2017	58	103	103	75mg/day	≥3 months	RFS: 1.002 (0.24-4.16)
									PFS:11.65 (0.11-1188.30)
Singla N (55)	USA	Cohort study; Prospective	2006-2012	73	203	99	81mg/day 325mg/day	NA	CSS: 3.14 (0.37-26.91)
									OS:1.91 (0.69-5.27)
									RFS:1.05 (0.64-1.74)
Pastore AL (56)	Italy	Cohort study; Retrospective	2008-2013	62	574	574	100mg/day	≥2 years	RFS: 0.74 (0.45-1.24)
Lipsky MJ (57)	USA	Cohort study; Retrospective	2001-2011	56-82	224	224	NA	NA	RFS: 2.41 (1.08-5.35)
Jacobs EJ (58)	USA	Cohort study; Prospective	1997-2008	NA	100139	302	Baby low dose; Adult strength dose	≥1 months	OS:0.75 (0.48-1.16)
Boorjian SA (59)	USA	Cohort study; Retrospective	1990-2006	57-75	907	907	NA	NA	RFS: 0.91 (0.751.10)
									PFS: 0.71 (0.52- 0.96)
Gee JR (60)	USA	Cohort study; Retrospective	1991-2003	67	43	43	81mg/day 325mg/day	NA	RFS:0.18 (0.06-0.52)
									PFS: 1.15 (0.81-1.63)
Ratnasinghe LD (61)	USA	Cohort study; Prospective	1982-1992	25-74	22794	40	NA	NA	OS: 3.36 (1.03-10.97)
This study	China	Case control; Retrospective	2002-2019	68	1121	1121	≥80mg/day	≥1 months	OS: 1.653 (0.871-3.137)
									RFS: 1.17 (0.772-1.774)

OS, Overall survival; CSS, Cancer-specific survival; PFS, Progression-free survival; RFS, Recurrence-free survival; MFS, Metastasis-free survival.

#### The Association Between Aspirin Intake and the Risk of Bladder Cancer

##### Overall Analysis

**Figure 3:**
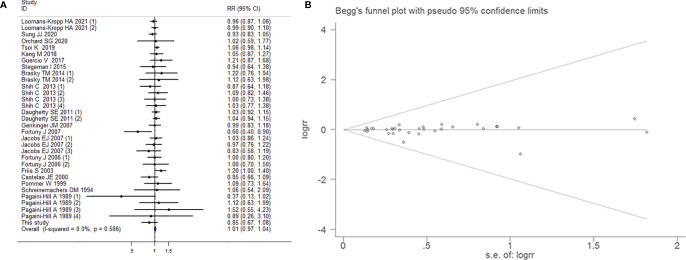
Forest plot **(A)** and funnel plot **(B)** showing the relationship between aspirin intake and the risk of bladder cancer in the overall analysis. The x-coordinate scale of solid lines perpendicular to the X-axis is 1. Each horizontal line segment parallel to the X-axis represents a confidence interval of the research results. The wider the confidence interval is, the longer the horizontal line segment. The small square in the middle of the horizontal line represents the position of the point estimate of the OR, and the size represents the weight of the study, which represents the percentage of the results of each study in the overall results. The intersection of the horizontal segment and the solid vertical line indicates that the study results are not statistically significant. Diamonds represent the overall effect of the estimate using the Mantel-Haenszel fixed-effects model. The visual examination of the funnel plot showed no apparent asymmetry, indicating that the publication bias was small and that the effect on the combined effect was negligible.

31 studies reported the effect estimates of the association between aspirin intake and the occurrence of BCa. The fixed-effects model was applied because the test for heterogeneity was not significant (I^2^=0.00%, *P*=0.586). We found that patients with aspirin intake did not have a significantly lower risk of BCa than those without aspirin intake (RR=1.007, 95% CI=0.975-1.041, *P*=0.671). The sensitivity analysis demonstrated that no single trial had significantly influenced the pooled RR (**Figure 3**). This result exhibited a low probability of publication bias, as determined by Begg's test (P=1.000) and Egger’s test (*P*=0.709, **Figure 3B**). However, low dose (<100mg/day) aspirin intake impacted on risk of BCa (RR=1.072, 95% CI=1.004-1.145, *P*=0.038).

##### Sensitivity Analysis

**Figure 4:**
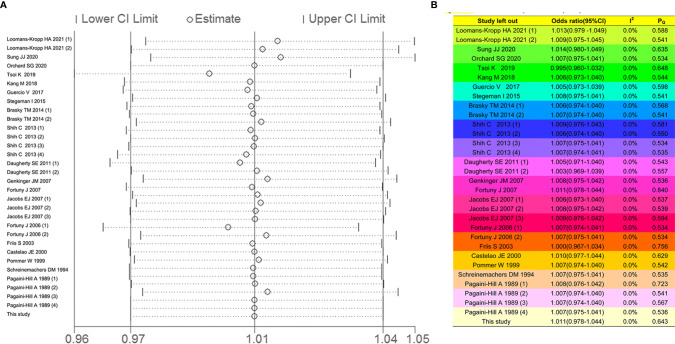
The leave-one-out sensitivity analysis for overall analysis. The forest plot **(A, B)** table showed the effects for heterogeneity of each study.

The effects for heterogeneity of each study were evaluated by leave-one-out sensitivity analysis. As shown in **Figure 4A**, the forest plots were equally applied, indicating that the identified studies in our meta-analysis had no significant influence to heterogeneity. Additionally, the respective values of *I^2^* and *P*_Q_ were enriched in **Figure 4B**. There were low heterogeneity after leaving the individual single study out, suggesting that the meta-analysis based on the pooled studies was credible.

##### Subgroup Analysis by Whether Regular Use of Aspirin

**Figure 5:**
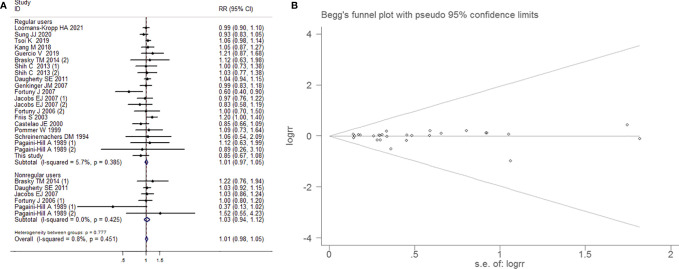
Forest plot **(A)** and funnel plot **(B)** showing the association of aspirin intake and the risk of bladder cancer in subgroup analysis by whether regularly intake aspirin.

Fixed-effects model was applied on account of little heterogeneity (I^2^=0.8%, *P*=0.451). There were no dependency between aspirin use and BCa in the group of regular users (RR=1.012, 95% CI=0.974-1.053, *P*=0.535) and nonregular users (RR=1.026, 95% CI=0.943-1.116, *P*=0.549), respectively. Begg’s test (*P*=0.802) and Egger’s funnel plot asymmetry test (*P*=0.624) manifested that there was no significant publication bias (**Figure 5**).

##### Subgroup Analysis by the Mean Duration of Use of Aspirin

**Figure 6:**
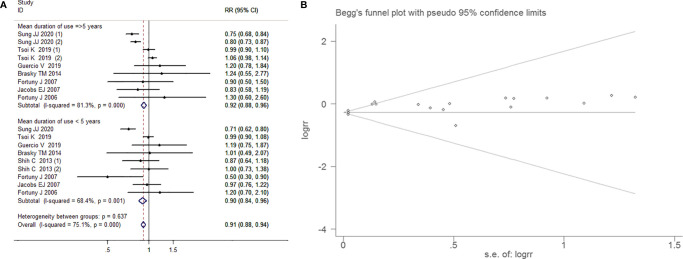
Forest plot **(A)** and funnel plot **(B)** showing the association of aspirin intake and the risk of bladder cancer in subgroup analysis by the mean duration of aspirin use.

Random effects model has been arranged because of strong heterogeneity (I^2^=75.1%, *P*<0.001). The crowd with a mean duration of use aspirin ≥5 years (RR=0.923, 95% CI=0.806-1.054, *P*=0.245) and even <5 years (RR= 0.901, 95% CI=0.771-1.06, *P*=0.192)were not associated with bladder cancer risk. There was no proof of significant publication bias by inspection of the funnel plot and formal statistical tests (Egger’s test, p=0.064; Begg’s test, *P*=0.272; **Figure 6**).

##### Subgroup Analysis by Sex

**Figure 7:**
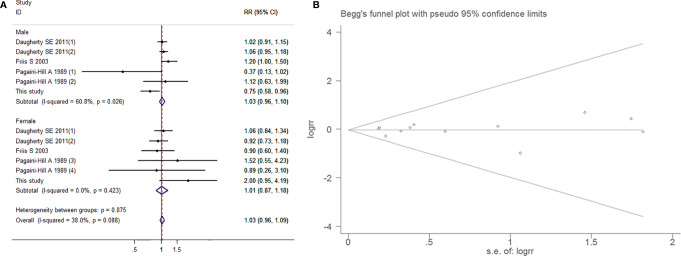
Forest plot **(A)** and funnel plot **(B)** showing the association of aspirin intake and the risk of bladder cancer in subgroup analysis by gender.

As a slight degree of heterogeneity was found (I^2^=38.0%, *P*=0.088), so a fixed-effects model was used. The results indicated no correlation between aspirin intake and BCa in either the male population (RR=1.028, 95% CI=0.958-1.102, *P*=0.449) or the female population (RR=1.014, 95% CI=0.873-1.178, *P*=0.856). Both Begg’s test (*P*=0.837) and Egger’s funnel plot asymmetry test (*P*=0.971) indicated that there was no significant publication bias (**Figure 7**).

##### Subgroup Analysis by Smoking Exposure

**Figure 8:**
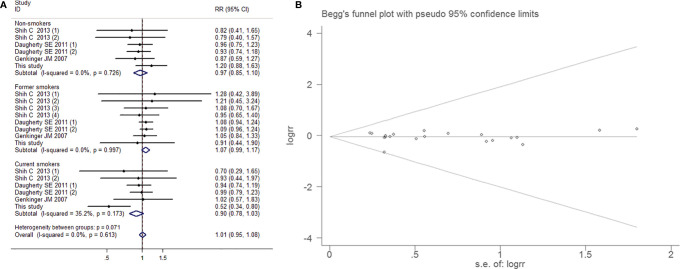
Forest plot **(A)** and funnel plot **(B)** showing the association of aspirin intake and the risk of bladder cancer in subgroup analysis by smoking status.

Smoking exposure in bladder cancer risk assessment is extremely necessary. There was no testimony for heterogeneity (I^2^=0.0%, *P*=0.613). There were no apparent pertinence of aspirin and BCa embodied in the groups of non-smokers (RR=0.966, 95% CI=0.845-1.104, *P*=0.610), former smokers (RR=1.073, 95% CI=0.989-1.165, *P*=0.091) and current smokers (RR=0.896, 95% CI=0.777-1.032, *P*=0.128), the overall analysis results also showed no difference(RR=1.012, 95% CI=0.951-1.078, *P*=0.707). Publication bias was not obvious (Egger’s test, *P*=0.817; Begg’s test, *P*=0.381; **Figure 8**).

##### Subgroup Analysis by Research Region

**Figure 9:**
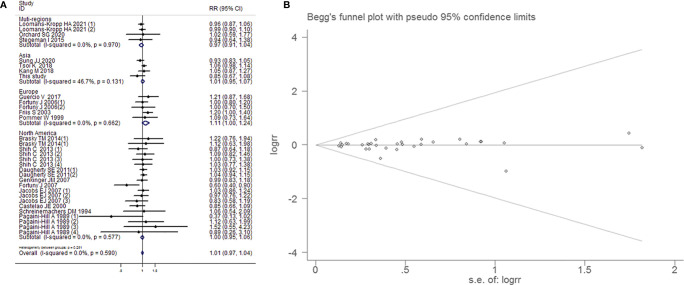
Forest plot **(A)** and funnel plot **(B)** showing the association of aspirin intake and the risk of bladder cancer in subgroup analysis by region.

Meta-analysis using a fixed-effects model (I^2^=0.0%, *P*=0.590) suggested that aspirin intake had no significant influence on the prevention of BCa in multi-regions (RR=0.974, 95% CI=0.910-1.043, *P*=0.456), Asian populations (RR=0.991, 95% CI=0.902-1.088, *P*=0.850), European populations (RR=1.112, 95% CI=0.996-1.242, *P*=0.059) and North American populations (RR=1.000, 95% CI=0.948-1.055, *P*=0.990). There was no evidence of significant publication bias by inspection of the funnel plot and formal statistical tests (Egger’s test, p=0.703; Begg’s test, *P*=0.961; **Figure 9**).

##### Subgroup Analysis by Study Type

**Figure 10:**
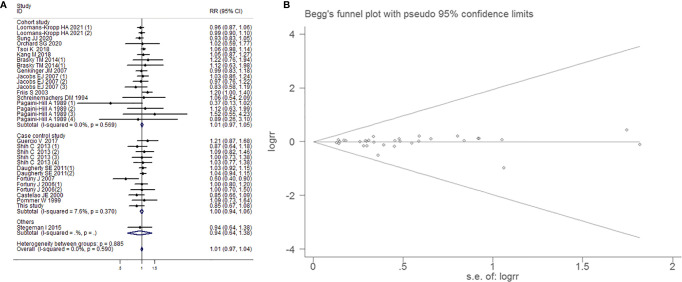
Forest plot **(A)** and funnel plot **(B)** showing the association of aspirin intake and the risk of bladder cancer in subgroup analysis by study type.

Within 18 cohort studies, aspirin intake was not linked to a decreased risk of BCa (RR=1.012, 95% CI=0.972-1.053, *P*=0.565). There was no evidence for heterogeneity (I^2^=0.0%, *P*=0.590). Moreover, when the data collected from 13 case-control studies (16, 17) were pooled (**Figure 10**), there was no statistically significant association between aspirin administration and BCa (RR=1.000, 95% CI=0.944-1.058, *P*=0.989). No association between aspirin intake and BCa was also examined in other study type subgroup (RR=1.007, 95% CI=0.975-1.041, *P*=0.752) Begg’s test (*P*=0.752) and Egger’s test (*P*=0.744) showed that the funnel plot distribution was symmetrical and that there was no significant publication bias.

#### The Association Between Aspirin Intake and Outcomes of Surgery for BCa

##### The Association Between Aspirin Intake and Intraoperative Conditions in Patients With BCa

Four studies suggested the relationship between aspirin intake and intraoperative conditions, including mean loss of hemoglobin, mean duration of operation and blood transfusion rate. The low and moderate heterogeneity was observed in blood transfusion rate group (I2=0.0%, P=0.814) and the mean duration of operation (I2=68.0%, P=0.08) group, using fixed-effects models; while high heterogeneity was determined in exploring the association between aspirin intake and the mean loss of hemoglobin (I2=81.0%, P=0.006) for BCa patients, and a random-effects model was applied. Compared with control group, aspirin intake had no impact on loss of hemoglobin with a combined MD of 0.08 (95% CI=-0.32-0.48, *P*=0.68) and the mean duration of operation with a combined MD of -2.36 (95% CI=-22.36-17.63, *P*=0.820). Moreover, there was no difference in blood transfusion rate during the resection of BCa between the aspirin group and the control group (RR=1.147, 95% CI=0.826-1.593, *P*=0.412).

##### The Association Between Aspirin Intake and Postoperative Complications in Patients With BCa

**Figure 11:**
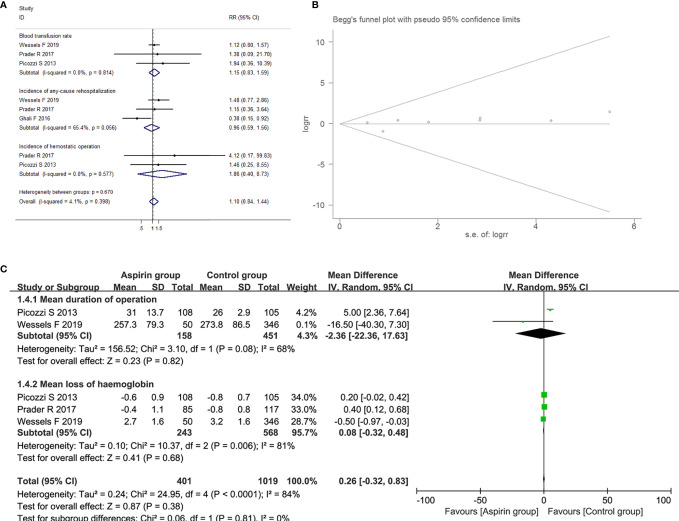
Forest plot **(A)** and funnel plot **(B)** showing the association of aspirin intake and the outcome of surgery, including blood transfusion rate, incidence of any-cause rehospitalization and the incidence of hemostatic operation; Forest plot **(C)** showing the association of aspirin intake and the outcome of surgery, including the mean duration of operation and mean loss of hemoglobin for BCa patients.

Three studies suggested the relationship between aspirin intake and postoperative complications including incidence of any-cause rehospitalization and hemostatic operation. As low heterogeneity was detected in any-cause rehospitalization (I2=65.4%, P=0.056) and hemostatic operation (I2=0.0%, P=0.577) groups, fixed-effects models were used. Compared to control group, aspirin intake had no impact on incidence of any-cause rehospitalization (RR=1.12, 95% CI=0.80-1.57, *P*=0.797) and incidence of hemostatic operation (RR=1.86, 95% CI=0.40-8.73, P=0.430). The above results showed in **Figures 11A–C**.

#### The Impact of Aspirin Intake on the Prognosis of Patients With Bladder Cancer

##### Aspirin Intake and the Survival of Patients With Bladder Cancer

**Figure 12:**
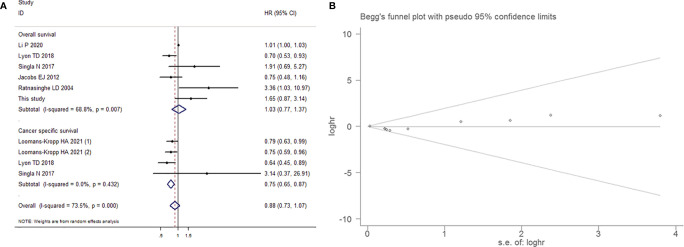
Forest plot **(A)** and funnel plot **(B)** showing the relationship between aspirin intake and the survival of patients with bladder cancer, which included overall survival and cancer-specific survival.

Among 11 articles and our study including a total of 8825 BCa patients with survival data, 6 studies investigated the relationship between aspirin intake and overall survival (OS) in BCa patients, and 4 studies reported cancer-specific survival (CSS). The random-effects model was used (**Figure 12** **)**, as higher heterogeneity was detected among these studies (*P*-value for heterogeneity<0.001; I^2^=73.5%). No significantly decreased levels of OS were observed in BCa patients who consumed aspirin compared with the controls, with a combined HR of 1.026 (95% CI=0.770-1.368, *P*=0.859). In contrast, aspirin intake had significant effects on the CSS of BCa patients (HR=0.749, 95% CI=0.646-0.869, *P*<0.001).

##### Aspirin Intake and Recurrence, Progression and Metastasis of Patients With Bladder Cancer

**Figure 13:**
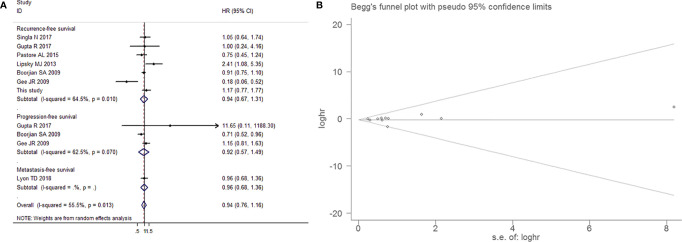
Forest plot **(A)** and funnel plot **(B)** showing the relationship between aspirin intake and recurrence-free survival and progression-free survival of patients with bladder cancer.

7 studies that reported the effect estimates of the association between aspirin intake and the recurrence, progression and metastasis of BCa. The random-effects model was applied, as the test for heterogeneity was significant (I^2^=55.5%, *P*=0.013). We found that patients with aspirin intake did not have a significantly lower risk of BCa recurrence (HR=0.936, 95% CI=0.667-1.313, *P*=0.701), progression (HR=0.922, 95% CI=0.570-1.489, *P*=0.739) and metastasis (HR=0.960, 95% CI=0.679-1.358, *P*=0.817) than those without aspirin intake, as shown in **Figure 13A**. This result exhibited a low probability of publication bias, as determined by Egger’s test (*P*=0.798, **Figure 13B**).

## Discussion

Aspirin is a non-steroidal anti-inflammatory drug with a long history. It is suitable for antipyretic and analgesic use and is widely used in the prevention of cardiovascular thrombosis (18). Recent studies have found that the long-term regular use of aspirin can significantly reduce the incidence of colorectal cancer, gastric cancer, liver cancer and other malignant tumors and improve the survival of patients with its anticancer effects (62–65). A population-based case-control study analyzed data from 1121 BCa cases and 2242 controls in New Hampshire and found that aspirin significantly reduced the risk of BCa, especially for tumors containing TP53 mutations (33). Similarly, Castelao J et al. (31) illustrated that there was a nonsignificant trend towards increased BCa risk in people with a longer duration of painkillers than in people who do not use painkillers, and regarding the effect of the correlation of different types of analgesics and BCa risk on the direction and strength of the obvious difference, aspirin showed weaker role. However, in a cohort study by Genkinger JM et al. (34). In the USA, there was no association between the frequency and dose of aspirin and BCa risk, which was not altered by known risk factors such as age, smoking or total fluid intake. To compensate for the lack of related research focusing on the population in Northeast China, a retrospective single-center case-control study analyzed data from 1121 BCa cases and 2242 controls in Northeast China was established to explore the association between the incidence of BCa and aspirin intake. Although we found several susceptibility factors were associated with strong risk of BCa, including gender (OR=1.263; *P*=0.028); marital status (OR=0.165; *P*<0.001); current smoking status (OR=2.360; *P*<0.001); current alcohol use (OR=0.615;*P*<0.001); former alcohol use(OR=0.314; *P*<0.001) and history of cardiovascular disease (OR=0.212; *P*<0.001), regular use of aspirin was not associated with a reduced risk of BCa (*P*=0.175). As studies of aspirin use and the risk of BCa have yielded mixed results, we further conducted meta-analyses and found that patients with aspirin intake did not have a significantly lower risk of BCa than those without aspirin intake after combining nineteen included articles and our study with more than 39524 BCa patients (*P*=0.671). Subgroup analysis by whether regular use of aspirin, by the mean duration of use of aspirin, by sex, by smoking exposure, by research region and by study type suggested that aspirin intake had no significant influence on the prevention of BCa.

Interestingly, in our case control study, subgroup analyses stratified by sex showed that there was no correlation between aspirin intake and the risk of BCa in female patients, however, the male population who regularly took aspirin had a lower incidence of BCa (OR=0.748; *P*=0.021). While considering the limited sample size and ethnic specificity, several explanations may help us better understand the above result. Androgens/Androgen Receptor (AR) system is considered as a central link to physiology and behaviors in male. Similarly, the development of clinical pathology of male malignant tumors could originate from this system (66). There were several studies identifying high expression of ARs in bladder cancer, suggesting that ARs could act as potential markers for monitoring and regulating malignant biological behaviors of BCa including metastasis, and recurrence (67, 68). The possible mechanisms contain DNA damage repair, promotion of p53 signaling, or activation of EGFR/ERBB2 pathway, etc. (69). As a mainstream therapy for advanced prostate cancer, androgen deprivation therapy (ADT) was founded having important potential in improving survival of bladder cancer, particularly non-muscle-invasive BCa (70). To our knowledge, antagonist of ARs, consisting of cyproterone, enzalutamide, docetaxel and so forth, is common medicine in ADT. Among these, as a potential application of ADT, aspirin could decrease the mRNA and protein expression of ARs, which has dose-dependence and time-dependence (71). Thereupon, we speculate that in male population, aspirin maybe significantly down-regulate ARs to inhibit the Androgens/Androgen Receptor system and subsequently suppress incidence of BCa, which need further molecular biological validation.

Meanwhile, it should be noted that subgroup analyses stratified by smoking status in our case control study found a significant reduction in the risk of BCa in current smokers with aspirin intake (OR=0.522; *P*=0.002), not in nonsmokers (*P*=0.252) or former smokers (*P*=0.807). Smoking and daily aspirin use are both risk factors for cancer. Some research indicates that aspirin usage in smokers is correlated with an increased risk of developing cancer, which could nullify the protective function of aspirin (72–74). Wang X et al. (73). found that aspirin use was related to a 29% lower CRC risk among never-smokers, compared to 17% lower CRC risk among smokers above median pack-years, indicating that heavy smokers could not be more favorable for protective role of aspirin against colorectal cancer than non- smokers. As cigarette was found to be more strongly associated with colorectal carcinoma which arise from serrated polyp pathway related to microsatellite instability, it was related to an increased risk of polyps in the left colon (75). While aspirin intake lowered the risk of serrated polyps in the right colon, indicating different subsites of colon cancer may be influenced by.aspirin and smoking (75). As playing role on platelet hyper-reactivity, smoking was strongly associated with increased risk of aspirin resistance, suggesting that the effect of aspirin may rely on independent carcinogenesis or progression (76) pathways of colorectal carcinoma among smokers and non-smokers. In breast cancer, cigarette smoking and aspirin/NSAID use interacted to affect breast cancer-specific mortality. Slattery ML et al. (76) reported that the strongest associations were observed for the interaction between cigarette smoking and aspirin intake with JAK/STAT signaling pathway including JAK2, STAT3, STAT5a, and STAT5b which were critical for cell development, cell proliferation, and apoptosis of breast cancer. As SOCS suppressed the signaling. Nicotine which were found in cigarette smoke condensates and activated the JAK2/STAT3 pathway (77), rs3816997 of SOSC2 may interacted with cigarette smoking and aspirin intake.

The prognostic value of aspirin use on monitor the intraoperative and postoperative outcome of BCa patients was also attracted increasing attention from scholars. After follow up 1121 BCa patients, we did not find that patients with a history of aspirin intake had a markedly longer overall or recurrence-free survival than those with no history of aspirin intake. Cox regression model did not indicate a history of aspirin intake was not an independent factor for overall or recurrence-free survival. Stratified analysis by sex showed no correlation between aspirin intake and the recurrence or survival of BCa for either male or female patients. Interestingly, we found that aspirin intake (HR=3.876; *P*=0.019) and type of surgery (HR=1.685; *P*=0.010) were significantly associated with overall survival in BCa patients younger than 68 years old. In the people who regularly used aspirin, majority of them has the history of coagulation abnormalities, consisting of platelet abnormalities and fibrinolysis (78–80). And coagulation abnormalities have long been closely related with the cardiovascular or cerebrovascular diseases, which are mostly found in the elderly and increased overall mortality considerably (81, 82). In addition, various of selection for statistics method may cause discrepancy the analysis outcomes.

Relative to our negative result about the effects of aspirin on tumor recurrence, a case-control study from Italy and a retrospective study from the USA showed a significant association between aspirin and recurrence in patients with BCa (55, 61). In the study of Gee JR (55), the 5-year RFS of aspirin users was 64.3%, which was significantly higher than that of non-aspirin users (26.9%), even after multivariate analysis adjusted for other factors. Considering the effect of aspirin on the survival rate of cancer patients, Lyon TD’s study (59) found that daily aspirin significantly improved survival outcomes after radical resection. It has also been observed that patients receiving low-dose aspirin had significantly better outcomes than patients receiving high-dose aspirin and those without aspirin. In contrast, a cohort study from the United States found no significant association between aspirin use and the overall survival of BCa patients (52). In addition, a prospective study by Singla N et al. (58) showed that the use of aspirin does not affect the survival of patients with any tumors, including CSS, OS, recurrence-free survival and cystectomy-free survival, regardless of the dose (81 or 325 milligrams a day). In response to these mixed findings, we performed a meta-analysis to investigate the impact of aspirin intake on the prognosis of patients with BCa and found that aspirin had no significant effects on the OS and CSS of BCa patients. In addition, patients with aspirin intake did not have a significantly lower risk of BCa recurrence, progression and metastasis than those without aspirin intake. We also found that there was no difference in blood transfusion rate, loss of hemoglobin, mean duration of operation, any-cause rehospitalization and incidence of any-cause rehospitalization during the resection of BCa between the aspirin group and the control group. Until now, available data have not supported a connection between aspirin exposure and the intraoperative situation and postoperative prognosis of BCa patients despite few prior studies.

Our limitations are as follows: one limitation is that this case-control study is a single-center study, which may lead to certain bias or heterogeneity. To the greatest extent possible to avoid such shortcomings or loopholes, we performed a meta-analysis to investigate the relationship between aspirin intake and the risk and prognosis of BCa. Some included studies did not provide available values of HR, RR or OR from multivariate analysis, so we performed calculations from the univariate data and compared them through formula calculations. The statistical methods we used cannot replace all the research methods. The conclusions we obtained without correlation are limited to the scope of our research, which may have some statistical errors (83, 84). Second, we only focused on researching patients who regularly consumed aspirin. Due to the limited available information from clinical data, stratification analysis according to different doses or durations was not conducted. The above factors may influence the results regarding the connection between the risk of BCa and the use of aspirin (10), which will be one of the focuses of our future research. Third, both our case-control study and meta-analysis showed no differences between aspirin intake and the risk and prognosis of BCa. However, previous *in vitro* studies have shown that aspirin may play a role as a chemopreventive agent in the OH-BBN/BDF BCa model (85), which suggests that there is heterogeneity between human epidemiological experiments and *in vitro* cell experiments.

## Conclusion and Outlook

Although aspirin has been predicted to be protective against various of tumors, differences in tumor origin and tissue specificity are bound to influence the results, which needed virtual simulated human microenvironment to explore whether aspirin can prevent or monitor BCa. Meanwhile, multi-center, large-sample, prospective studies are also needed. Advances in cancer biology clearly indicate that the development of malignant tumors is the result of complex interactions and the integration of gene expression, proteome and metabolome changes, involving genetics, microbes, diet, drugs (86, 87), and other factors such as different living conditions, exposure factors and ethnic differences (88). In the age of precision oncology, individual cancer treatment should be personalized based on genetic and environmental factors. In addition, aspirin, as a sensitizer, can increase the therapeutic effect of chemotherapy for cancers of the colon and stomach, while there are few studies in the urinary system, which will be a promising research direction. As a continuation of this study, it is necessary to further explore pathological examinations, immune parameters, tumor molecular markers and other aspects from the database, which contributes greatly to the development of BCa treatment.

## Data Availability Statement

The datasets generated for our case-control study are available on request to the corresponding authors. Additionally, publicly available datasets for meta-analysis in our study could be searched in the PubMed, Embase, Ovid Medicine, Cochrane Library and Scopus databases.

## Ethics Statement

This study was approved by the institutional review committee of the Second Affiliated Hospital of Dalian Medical University and written informed consent was obtained from each patient or their next of kin. (Approval Ref. No. KY2018-120)

## Author Contributions

BF, XL, and GW contributed to the study concept and design, undertook project leadership and guaranteed this work. YH, HL, HJ, MFS, ST, WX, QL, TH, ZY, and RZ were responsible for data collection and interpretation. ZY and RZ controlled quality of data and algorithms. BF, AM, YH, HJ, and ST analyzed data and interpreted the data. BF, GW, HL, HJ, and MS wrote the first draft of the manuscript. BF, HY, and AM reviewed and revised the manuscript. All authors contributed to the article and approved the submitted version.

## Funding

This study was supported by the National Natural Science Foundation of China (81972831 and 31800787),the United Fund of the Second Hospital of Dalian Medical University and Dalian Institute of Chemical Physics, Chinese Academy of Sciences (UF-QN-202004), the Dalian High-level Talents Innovation Support Program (2019RQ014) and the Doctoral Research Startup Foundation of the Second Hospital of Dalian Medical University (DY2Y201704).

## Conflict of Interest

The authors declare that the research was conducted in the absence of any commercial or financial relationships that could be construed as a potential conflict of interest.
